# Effect of image quality fluctuations on the repeatability of thickness measurements in swept-source optical coherence tomography

**DOI:** 10.1038/s41598-020-70852-y

**Published:** 2020-08-17

**Authors:** Heon Yang, Hye Sun Lee, Hyoung Won Bae, Gong Je Seong, Chan Yun Kim, Sang Yeop Lee

**Affiliations:** 1 Kong Eye Center, Seoul, Republic of Korea; 2 Department of Ophthalmology, Institute of Vision Research, Severance Hospital, Yonsei University College of Medicine, Seoul, Republic of Korea; 3 Department of Ophthalmology, Yongin Severance Hospital, Yonsei University College of Medicine, Yongin, Gyeonggi-do Republic of Korea; 4 Biostatistics Collaboration Unit, Department of Research Affairs, Yonsei University College of Medicine, Seoul, Republic of Korea

**Keywords:** Diseases, Eye diseases, Optic nerve diseases, Medical research, Outcomes research

## Abstract

This study investigated the effect of image quality fluctuations on the repeatability of thickness measurements of the peripapillary retinal nerve fibre (PP-RNFL) and ganglion cell-inner plexiform (GC-IPL) layers using swept-source optical coherence tomography (SS-OCT). Three consecutive OCT scans each were performed on 56 healthy subject. Finally, 168 SS-OCT results were analysed. Based on the tertile values of the mean absolute difference of image quality score, all subjects were divided into the following three groups—low-(LIQD), moderate-(MIQD), and high-(HIQD) image quality score difference groups. A linear mixed model and intraclass correlation coefficients (ICCs) were used for analyses. Despite high ICC values (> 0.9), several sectors showed significant differences in the ICC values in intergroup comparisons. For LIQD-HIQD and MIQD-HIQD, most PP-RNFL sectors showed significant differences. For GC-IPL sectors, the LIQD-HIQD comparison showed significant differences in the temporosuperior (*p* = 0.012), inferior (*p* < .001), and temporoinferior (*p* = 0.042) sectors. Significant differences existed in the average GC-IPL (*p* = 0.009), nasoinferior (*p* = 0.035), and inferior GC-IPL sectors (*p* < .001) for MIQD-HIQD comparison. With higher image quality fluctuations, the repeatability of SS-OCT decreased in several sectors, which are considered clinically relevant in evaluating glaucoma status. Therefore, maintaining high-quality image status is essential to enhance the reliability of SS-OCT.

## Introduction

Optical coherence tomography (OCT) is an indispensable ophthalmic imaging technology that effectively identifies retinal structural alterations. OCT technologies have undergone longitudinal development from time-domain OCT to spectral-domain OCT (SD-OCT) and swept-source OCT (SS-OCT). The recently developed SS-OCT uses a tunable light source with a central wavelength of 1,050 nm, and a photodiode detector with a semiconductor camera for light detection. These features permit a high scanning speed and a deep imaging range with uniform sensitivity. In glaucoma cases, these technological advances in OCT device have eased the measurement of changes in the peripapillary retinal nerve fibre layer (PP-RNFL) and ganglion cell-inner plexiform layer (GC-IPL) thickness. Both these layers are critical to evaluate the extent of damage of the glaucomatous optic nerve.

Diagnostic precision is of utmost importance when diagnosing a disease or monitoring its progression using OCT. Both image quality and repeatability/reproducibility of an OCT measurement affect its overall diagnostic precision. Segmentation error and misalignment of measurement area generate artefacts that affect the image quality and, ultimately, the OCT measurement values^1–3^. Repeatability and reproducibility relate to the scatter of measured values and indicate whether a constant value is obtained when the same object is measured repeatedly. These parameters are helpful to monitor disease progression because repeated measurements are performed over time at the same anatomical region of an individual patient. Both SD-OCT and SS-OCT have demonstrated repeatability and reproducibility for clinical use^4–7^, which is an important reason for the widespread use of OCT in the diagnosis and management of various ocular conditions, including glaucoma.

Although image quality and repeatability/reproducibility of OCT images are important factors when interpreting the results, only few studies have previously investigated the effect of image quality fluctuations on repeatability or reproducibility^8,9^. Moreover, these studies were implemented using time-domain OCT or SD-OCT at the peripapillary area. Therefore, this study aimed to evaluate the effect of image quality fluctuations on the repeatability of SS-OCT measurement values in both the macular and peripapillary areas. The results of this study indicate the importance of maintaining image quality in SS-OCT while performing repeated measurements.

## Results

Of the 58 healthy subjects who were selected for OCT imaging, two were excluded based on their image quality scores. SS-OCT data of 56 subjects (25 men and 31 women), comprising 168 results from the three consecutive OCT examinations, were analysed. Based on the tertile values of the mean absolute difference of image quality score, the subjects were stratified into three groups—low image quality score difference group (LIQD; with scores ranging between 0.06 and 0.86 in PP-RNFL, and between 0.067 and 0.747 in GC-IPL), moderate image quality score difference group (MIQD; with scores ranging between 0.873 and 1.927 in PP-RNFL, and between 0.753 and 1.227 in GC-IPL), and high image quality score difference group (HIQD; with scores ranging between 1.947 and 10.053 in PP-RNFL, and between 1.253 and 8.3 in GC-IPL). The three groups showed no significant differences in their demographic or clinical characteristics (Table 1).

**Table 1:** Comparison of demographics and clinical characteristics among groups.

	All subjects	LIQD	MIQD	HIQD	*p* value*
Age, years	53.31 ± 15.92	51.52 ± 14.89	54.71 ± 11.36	53.83 ± 17.59	0.325
Sex (M:F)	26:30	10:8	7:12	9:10	0.223
Central corneal thickness, µm	542.22 ± 33.72	538.37 ± 28.64	542.79 ± 31.45	545.54 ± 23.61	0.641
Spherical equivalent, D	1.35 ± 2.17	0.87 ± 3.36	1.42 ± 2.26	1.76 ± 1.14	0.623
Axial length, mm	23.17 ± 1.2	23.05 ± 1.17	23.11 ± 1.52	23.35 ± 0.77	0.594
Intraocular pressure, mmHg	14.42 ± 2.84	13.82 ± 3.46	15.56 ± 2.63	13.88 ± 2.44	0.076

*LIQD* low image quality difference group, *MIQD* moderate image quality difference group, *HIQD* high image quality difference group, *SD* standard deviation.

*Analysis of variance or chi-square test; all values are represented as mean ± SD or ratio.

### Comparison of PP-RNFL and GC-IPL thicknesses among the three groups

Table 2 shows results for the comparison of PP-RNFL and GC-IPL thicknesses among the three groups at each measurement sector. The linear mixed model showed no significant differences in PP-RNFL and GC-IPL thicknesses of different sectors among the three groups (Table 2). However, when the difference in image quality between OCT examinations was large, GC-IPL tended to be thick; this tendency was not seen in the peripapillary sectors.

**Table 2:** Comparison of thickness values measured using SS-OCT among the three groups.

	LIQD	MIQD	HIQD	Overall p*
PPAver	105.76 ± 2.01	105.95 ± 1.96	109.67 ± 1.96	0.294
4 T	78.96 ± 2.84	80.66 ± 2.77	77.33 ± 2.77	0.697
4 S	136.21 ± 3.258	136.4 ± 3.17	136.3 ± 3.17	0.999
4 N	70.04 ± 3.788	70.59 ± 3.687	74.99 ± 3.69	0.589
4 I	136 ± 3.284	136.37 ± 3.197	144.49 ± 3.19	0.117
12 T	66.779 ± 2.26	67.612 ± 2.2	66.69 ± 2.2	0.948
12 TS	92.523 ± 3.395	92.407 ± 3.305	95.93 ± 3.31	0.698
12 ST	141.94 ± 5.299	143.21 ± 5.158	144.86 ± 5.16	0.925
12 S	142.89 ± 5.881	143.9 ± 5.724	137.94 ± 5.72	0.736
12 SN	125.81 ± 5.51	124.17 ± 5.363	126.54 ± 5.36	0.95
12 NS	80.382 ± 4.685	79.865 ± 4.56	81.61 ± 4.56	0.962
12 N	59.553 ± 2.879	59.097 ± 2.802	62.89 ± 2.8	0.584
12 NI	69.555 ± 4.568	71.495 ± 4.446	78.49 ± 4.45	0.339
12 IN	114.12 ± 4.993	106.72 ± 4.86	114.37 ± 4.86	0.458
12 I	150.88 ± 5.699	143.38 ± 5.547	158.22 ± 5.55	0.177
12 IT	144.88 ± 6.09	155.52 ± 5.927	151.35 ± 5.93	0.458
12 TI	78.132 ± 3.722	80.993 ± 3.623	76.8 ± 3.62	0.707
GCLAver	69.604 ± 1.206	70.089 ± 1.174	71.402 ± 1.174	0.544
TS	71.632 ± 1.215	72.165 ± 1.183	72.685 ± 1.183	0.825
S	68.888 ± 1.287	68.93 ± 1.252	70.747 ± 1.252	0.496
NS	71.983 ± 1.44	72.076 ± 1.402	75.057 ± 1.402	0.224
NI	69.546 ± 1.379	69.868 ± 1.342	71.046 ± 1.342	0.713
I	64.464 ± 1.181	65.254 ± 1.149	65.903 ± 1.149	0.685
TI	71.16 ± 1.358	73.046 ± 1.322	73.133 ± 1.322	0.508

*SS-OCT* swept-source optical coherence tomography, *LIQD* low image quality difference group, *MIQD* moderate image quality difference group, *HIQD* high image quality difference group, *SE* standard error, *PPAver* average PP-RNFL thickness, *T* temporal, *S* superior, *N* nasal, *I* inferior, *TS* temporosuperior, *ST* superotemporal, *SN* superonasal, *NS* nasosuperior, *NI* nasoinferior, *IN* inferonasal, *IT* inferotemporal, *TI* temporoinferior, *GCLAver* average GC-IPL thickness.

*Linear mixed model; all values are represented as least-squares mean ± SE.

### Correlations between image quality and SS-OCT results at each measurement sector

Correlation analyses between image quality and OCT results at each measurement sector were performed for repeated measurements (Table 3). After adjusting for age and sex, five sectors showed significant negative correlations between image quality and PP-RNFL (average PP-RNFL, superotemporal, superior, inferior, and temporoinferior sectors) or GC-IPL (average GC-IPL, temporosuperior, nasoinferior, inferior, and temporoinferior sectors).

**Table 3:** Correlations and partial correlations between image quality and SS-OCT results.

	Correlation*		Partial correlation^†^	
	R	*p*	r	*p*
PPAver	− 0.258	**0.001**	− 0.225	**0.004**
4 T	− 0.054	0.489	− 0.089	0.254
4 S	− 0.151	**0.049**	− 0.147	0.058
4 N	− 0.144	0.062	− 0.061	0.436
4 I	− 0.135	0.08	− 0.113	0.147
12 T	0.02	0.795	− 0.012	0.878
12 TS	0.031	0.691	0.025	0.753
12 ST	− 0.137	0.077	− 0.169	**0.029**
12 S	− 0.156	**0.043**	− 0.155	**0.047**
12 SN	0.039	0.619	0.071	0.364
12 NS	− 0.087	0.263	− 0.026	0.738
12 N	0.016	0.834	0.129	0.099
12 NI	− 0.178	**0.021**	− 0.094	0.227
12 IN	− 0.191	**0.013**	− 0.148	0.058
12 I	− 0.187	**0.015**	− 0.175	**0.024**
12 IT	− 0.018	0.821	− 0.03	0.697
12 TI	0.254	**0.001**	0.234	**0.002**
GCLAver	− 0.176	**0.023**	− 0.186	**0.017**
TS	− 0.181	**0.019**	− 0.175	**0.025**
S	− 0.113	0.146	− 0.123	0.114
NS	− 0.115	0.137	− 0.134	0.086
NI	− 0.169	**0.029**	− 0.189	**0.015**
I	− 0.192	**0.013**	− 0.207	**0.008**
TI	− 0.193	**0.012**	− 0.19	**0.014**

*SS-OCT* swept-source optical coherence tomography, *PPAver* average PP-RNFL thickness, *T* temporal, *S* superior, *N* nasal, *I* inferior, TS, temporosuperior, *ST* superotemporal, *SN* superonasal, *NS* nasosuperior, *NI* nasoinferior, *IN* inferonasal, *IT* inferotemporal, *TI* temporoinferior, *GCLAver* average GC-IPL thickness.

*Pearson’s correlation estimated using a linear mixed model; ^†^Partial correlation after age- and sex-adjustments; significant *p* values are shown as bold-face text.

### Comparisons of repeatability among the three groups at each measurement sector

**Figure 1:**
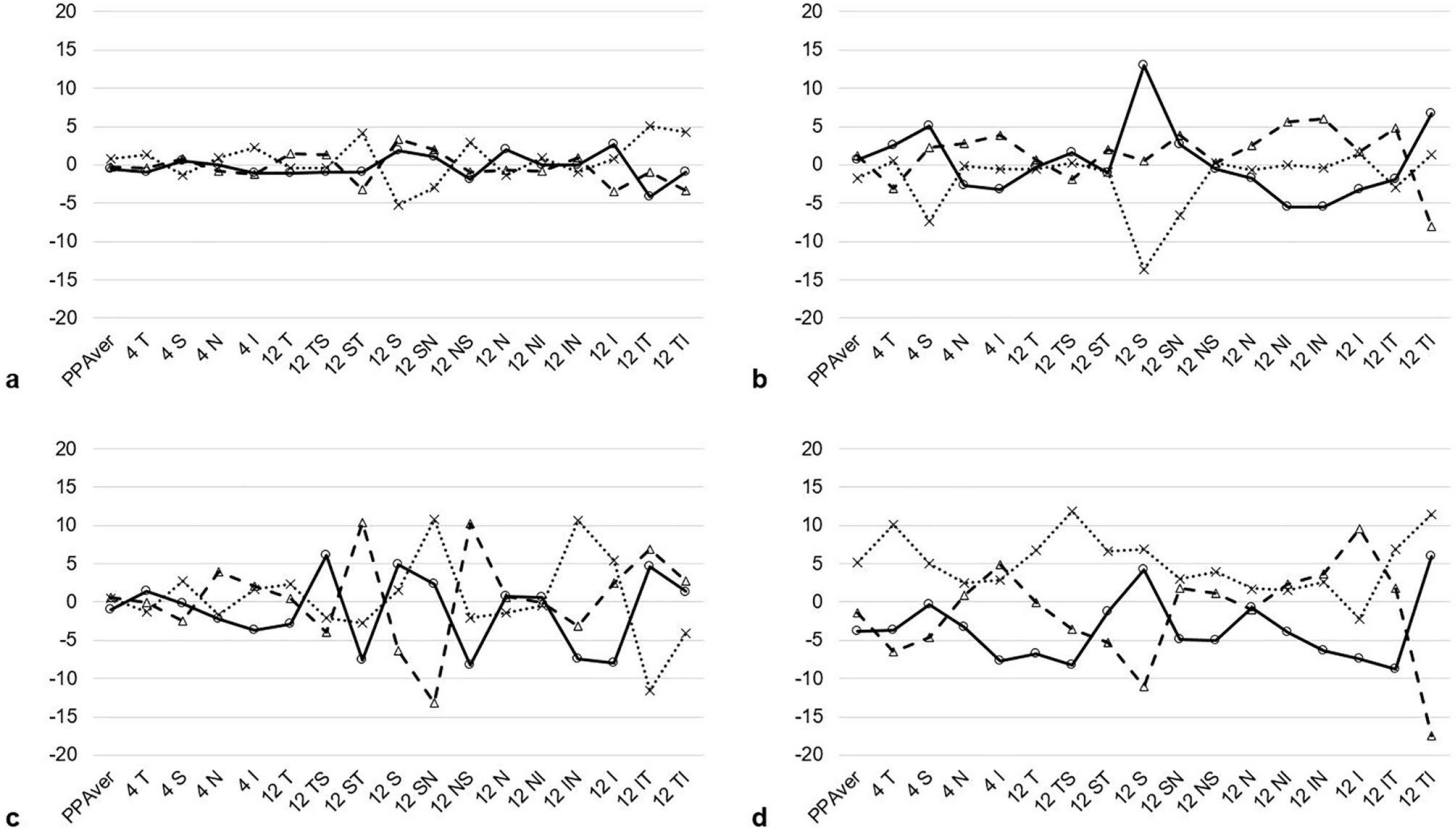
The representative results of differences between the measured values by image quality difference at each sector of PP-RNFL. The image quality difference for a, b, c, and d was 0.107, 0.88, 5.233, and 8.087, respectively. X-axis indicates the measurement sectors, and Y-axis indicates the difference between the masured values. The solid line indicates the difference between the first and second measurements. The thick dotted line indicates the difference between the second and third measurements. The thin dotted line indicates the difference between the first and third measurements. *PP Aver* average PP-RNFL thickness, *T* temporal, *S* superior, *N* nasal, *I* inferior, *TS* temporosuperior, *ST* superotemporal, *SN* superonasal, *NS* nasosuperior, *NI* nasoinferior, *IN* inferonasal, *IT* inferotemporal, *TI* temporoinferior, *PP-RNFL* peripapillary retinal nerve fibre layer.

ICC of three consecutive measurement values was calculated and compared among the groups (Table 4). The overall repeatability was high in all sectors for all groups (ICC > 0.8). The ICC values were the lowest for the HIQD group in every measurement sector. Figure 1 shows the representative results for difference in thickness at each measurement sectors of PP-RNFL by image quality difference. With increase in the image quality difference value, the difference between the measured values increased accordingly. Results of between-group comparisons showed significant differences in repeatability at only two sectors (temporoinferior for PP-RNFL; inferior for GC-IPL) in the LIQD and MIQD groups. In addition, results of comparisons between LIQD and HIQD groups, and between MIQD and HIQD groups, showed significant differences in repeatability at most sectors for PP-RNFL, except at the superior, nasal, superior nasal, and nasoinferior sectors. On comparison of repeatability in GC-IPL sectors, significant differences were seen at the temporosuperior, inferior, and temporoinferior sectors between LIQD and HIQD groups, and at the average GC-IPL, nasoinferior, and inferior sectors between MIQD and HIQD groups. No sector showed significant differences in repeatability when compared between LIQD and MIQD groups. The proportion of sectors affected by image quality fluctuations was higher in PP-RNFL than in GC-IPL.

**Table 4:** Comparison of repeatability among the three groups.

	LIQD n = 18	MIQD n = 19	HIQD n = 19	*p* value*		
				LIQD versus MIQD	LIQD versus HIQD	MIQD versus HIQD
PPAver	0.996 (0.992–0.999)	0.996 (0.992–0.998)	0.964 (0.925–0.985)	> .999	**0.002**	**0.001**
4 T	0.996 (0.992–0.998)	0.996 (0.992–0.998)	0.982 (0.962–0.992)	> .999	**0.03**	**0.028**
4 S	0.99 (0.978–0.996)	0.988 (0.974–0.995)	0.978 (0.954–0.991)	0.792	0.254	0.373
4 N	0.996 (0.99–0.998)	0.998 (0.996–0.999)	0.993 (0.985–0.997)	0.319	0.421	0.067
4 I	0.989 (0.976–0.995)	0.99 (0.978–0.996)	0.956 (0.906–0.982)	0.891	**0.044**	**0.029**
12 T	0.995 (0.99–0.998)	0.996 (0.992–0.998)	0.956 (0.906–0.982)	0.748	**0.002**	**< .001**
12 TS	0.99 (0.978–0.996)	0.994 (0.987–0.997)	0.956 (0.907–0.982)	0.462	**0.031**	**0.003**
12 ST	0.977 (0.95–0.991)	0.995 (0.989–0.998)	0.878 (0.741–0.949)	**0.028**	**0.014**	**< .001**
12 S	0.993 (0.984–0.997)	0.973 (0.944–0.989)	0.965 (0.927–0.986)	0.051	**0.02**	0.701
12 SN	0.98 (0.957–0.992)	0.987 (0.973–0.995)	0.964 (0.923–0.985)	0.533	0.392	0.133
12 NS	0.985 (0.967–0.994)	0.996 (0.992–0.998)	0.966 (0.928–0.986)	0.057	0.235	**0.002**
12 N	0.997 (0.993–0.999)	0.997 (0.993–0.999)	0.899 (0.784–0.958)	> .999	**< .001**	**< .001**
12 NI	0.995 (0.989–0.998)	0.99 (0.979–0.996)	0.981 (0.959–0.992)	0.318	0.054	0.346
12 IN	0.976 (0.948–0.99)	0.988 (0.975–0.995)	0.88 (0.745–0.95)	0.316	**0.017**	**< .001**
12 I	0.987 (0.972–0.995)	0.992 (0.983–0.997)	0.965 (0.925–0.985)	0.484	0.151	**0.03**
12 IT	0.99 (0.979–0.996)	0.972 (0.941–0.988)	0.869 (0.721–0.945)	0.136	**< .001**	**0.02**
12 TI	0.996 (0.992–0.998)	0.984 (0.967–0.994)	0.829 (0.637–0.929)	**0.046**	**< .001**	**< .001**
GCLAver	0.997 (0.994–0.999)	0.999 (0.998–1)	0.994 (0.986–0.997)	0.115	0.319	**0.009**
TS	0.997 (0.993–0.999)	0.991 (0.98–0.996)	0.983 (0.964–0.993)	0.114	**0.012**	0.351
S	0.998 (0.996–0.999)	0.996 (0.992–0.998)	0.993 (0.985–0.997)	0.329	0.072	0.413
NS	0.999 (0.997–0.999)	0.998 (0.996–0.999)	0.997 (0.994–0.999)	0.319	0.115	0.554
NI	0.995 (0.99–0.998)	0.997 (0.993–0.999)	0.987 (0.972–0.995)	0.456	0.168	**0.035**
I	0.997 (0.993–0.999)	0.994 (0.986–0.997)	0.93 (0.851–0.971)	0.319	**< .001**	**< .001**
TI	0.994 (0.986–0.997)	0.986 (0.969–0.994)	0.976 (0.949–0.99)	0.222	**0.042**	0.435

*ICC* intraclass correlation coefficient, *CI* confidence interval, *LIQ* low image quality difference group, *MIG* moderate image quality difference group, *HIQ* high image quality difference group, *LSM* least-squares mean, *SE* standard error, *PPAver* average PP-RNFL thickness, *T* temporal, *S* superior, *N* nasal, *I* inferior, *TS* temporosuperior, *ST* superotemporal, *SN* superonasal, *NS* nasosuperior, *NI* nasoinferior, *IN* inferonasal, *IT* inferotemporal, *TI* temporoinferior, *GCLAver* average GC-IPL thickness.

*Z-test, data are represented as ICC (95% CI); significant *p* values are shown as bold-faced text.

## Discussion

The results of this study, which investigated the association between image quality fluctuations and repeatability of SS-OCT measurements, showed that repeatability decreases with an increase in image quality fluctuation in several sectors of PP-RNFL and GC-IPL. These observations were made in healthy subjects with an OCT image quality > 60, which was calculated as per manufacturer’s recommendation for clinical use. Therefore, it can be said that our study was conducted under settings wherein the factors affecting OCT results, such as low image quality (image quality score < 60) and structural alteration by ocular disease, were controlled. In addition, when the study groups were compared based on the mean absolute difference among three consecutive OCT measurements, no significant differences were noted in the measured thickness at any of the measurement sectors (Table 2). This result also indicates that there was no large deviation in the measured values of our data set. Nevertheless, even with good image quality (recommended for clinical use) and high repeatability (based on ICC), the measurement repeatability was affected by image quality fluctuations in several sectors, especially in comparisons involving the HIQD group. Moreover, this phenomenon affected sectors that are considered important in glaucoma management. Thus, it is crucial to maintain not only a high level of image quality but also a constant value of image quality for the clinical application of SS-OCT.

Interestingly, although the HIQD group had the lowest ICC value of each measurement sector among the three groups, not all sectors showed significant differences on comparison with the LIQD or MIQD groups. In addition, only five sectors of the clock-hour map for PP-RNFL (superotemporal, nasal, inferonasal, inferotemporal, and temporoinferior sectors) showed ICC values under 0.9. If repeatability is exclusively determined by image quality, the repeatability of the OCT results obtained from subjects of HIQD group should be lower regardless of location of the measurement sectors. Segmentation is important for analysing the thickness of the retinal layer using OCT results. Although image quality is a critical factor for segmentation, ocular structural factors such as axial length, shape of optic disc, or tortuosity of retinal vessel also affect segmentation^3,10,11^. The superotemporal, inferonasal, inferotemporal, and temporoinferior sectors contain retinal blood vessels, which contribute to the structural variation of the parapapillary area. Thus, the anatomic structure around the optic disc, which varies largely even in healthy eyes, could have influenced the repeatability.

Inter-individual diversity in the optic disc shape and peripapillary structures contribute to inaccuracies in the measurement of PP-RNFL thickness by OCT. In contrast, the macular area is well-known for its inter-individual similarities^12–14^. Such inaccuracies might influence clinical decision-making in glaucoma management. Therefore, several studies have emphasised on the usefulness of GC-IPL parameters for the diagnosis of glaucoma in myopic eyes^15–17^. In the present study, the repeatability of GC-IPL sectors was relatively less affected by image quality fluctuations as compared to PP-RNFL sectors. This result further supports the usefulness of macular GC-IPL thickness evaluation for estimating glaucoma status, although further studies on patients with glaucoma are required to confirm this occurrence. Previous studies have shown a positive correlation between image quality and OCT-based measurement of macular or PP-RNFL thickness^18–21^, i.e., a reduction in image quality decreases the macular or PP-RNFL thickness, thereby leading to incorrect OCT interpretations of glaucoma progression. In this study, image quality correlated significantly in several sectors for both PP-RNFL and GC-IPL thickness, and this result did not change even after adjusting for age and sex. Therefore, image quality remains an essential factor in the interpretation of SS-OCT results. Unlike the correlation results reported previously, the negative correlation between the thickness values and image quality may be due to repeated measurements, small sample size, or unknown intrinsic characteristics of SS-OCT. It is possible that a study on patients with glaucoma may yield negative correlation between the thickness values and image quality.

Studies on the relationship between image quality fluctuations and repeatability of OCT measurements are limited. Lee et al. reported the effect of signal strength difference on the repeatability of PP-RNFL thickness in time-domain OCT^8^, and Kim et al. reported the effect of signal strength on PP-RNFL thickness and colour-coded classification in SD-OCT^9^. Both studies inferred that substantial differences in the signal strength lower the repeatability. Our study presents similar results using SS-OCT. Compared to previous studies, the use of three consecutive measurements for statistical analysis provide more reliability to this study, and this strategy is more appropriate for identifying the impact of image quality fluctuation on OCT results.

This study has several limitations. First, although the data were collected prospectively, the number of subjects included was relatively small. Second, the effect of image quality fluctuation on repeatability was studied in healthy subjects. A similar study on patients with glaucoma will help to understand the clinical significance of image quality fluctuations on SS-OCT results. Third, the results of our study cannot be applied directly to other studies focused on other types of OCT. This is because the image quality score which was used for calculating image quality fluctuation in the present study was developed by the manufacturer of DRI OCT, although it is not difficult to predict that the accuracy of segmentation of the OCT will be lowered if the quality of the image deteriorates. Further studies involving other types of OCT seem necessary to clarify the effect of image quality fluctuation on repeatability in each type of OCT. Despite these limitations, our findings are meaningful because this is the first study to investigate the effect of image quality fluctuation on repeatability in SS-OCT using prospectively collected data.

In conclusion, this study reported that higher image quality fluctuation leads to lower repeatability of SS-OCT results in several sectors of PP-RNFL and GC-IPL. Interestingly, the identified sectors were clinically important for glaucoma management. In addition, the repeatability of GC-IPL sectors was relatively less affected than that of PP-RNFL sectors by image quality fluctuations. Thus, maintaining a high-quality image status is vital to enhance the reliability of SS-OCT for PP-RNFL and GC-IPL measurements, more so in the PP-RNFL region.

## Methods

This study collected raw data retrospectively from the dataset used in a previous study to compare the repeatability and agreement between SD-OCT and SS-OCT in healthy eyes^5^. The institutional review board of Yonsei University Severance Hospital, Seoul, Korea, approved this study (1-2019-0043), and the need for written informed consent was waived because of the retrospective study design. The study adhered to the tenets of the Declaration of Helsinki. The detailed characteristics of the subjects in dataset have been described previously^5^. Normal subjects who had visited the glaucoma clinic at our hospital between August 2014 and December 2014 were enrolled Medical history, Snellen best-corrected visual acuity (BCVA), slit-lamp biomicroscopy findings, intraocular pressure (IOP; Goldmann applanation tonometry), and indirect ophthalmoscopy findings were obtained. In addition, the following data were acquired: axial length estimated using the IOL Master (Carl Zeiss Meditec AG, Jena, Germany); central corneal thickness calculated using ultrasound pachymetry (DGH-1000; DGH Technology Inc., Frazer, PA, USA); optic disc and RNFL thickness measurements performed using a + 90 diopter (D) lens, colour disc, and red-free photography (VISUCAM200, Carl Zeiss Meditec AG, Jena, Germany). Optic nerve function had been estimated using a Humphrey Visual Field analyser (24-2 Swedish Interactive Threshold Algorithm; Carl Zeiss Meditec, Inc., Dublin, CA, USA).

Healthy subjects of age > 19 years with a BCVA ≥ 20/25 and no evidence of glaucomatous optic disc changes, RNFL defects, or visual field changes with IOP < 21 mmHg were included retrospectively. The eye that was analysed in each patient was selected randomly. Exclusion criteria were the presence of cataract grade of Lens Opacities Classification System III > 3, axial length > 24.5 mm, refractive errors with spherical equivalent >  ±5D, or cylindrical error >  ±3D, and any medical or ophthalmic conditions that influenced the optic disc, RNFL, and visual field measurements.

### Thickness measurement using SS-OCT for repeatability

In this study, we used the DRI OCT-1 system (Topcon, Tokyo, Japan, analysis software version 9.1.2.28693), which had a high-speed wavelength tuning laser source with central wavelength of 1,050 nm. This SS-OCT system had an image acquisition speed of 100,000 A-scan/second, with an axial and transverse resolutions of 8 and 20 µm, respectively. Three consecutive SS-OCT scans were acquired on the same day with an interval of at least 5 min between the scans. A single technician performed all scans using an internal fixation target. Pupillary dilation was performed in all subjects. A three-dimensional (3D) optic disc and 3D wide scan protocols were used to measure PP-RNFL and GC-IPL thicknesses, respectively. The 3D optic disc scan covered a 6 × 6-mm area on the optic disc and comprised 512 A-scans × 256 B-scans. PP-RNFL thickness was measured in a 3.4-mm-diameter scan circle centred on the optic disc. The 3D wide scan protocol covered a 12 × 9-mm rectangular area centred between the optic disc and fovea and comprised 512 A-scans × 256 B-scans. PP-RNFL thicknesses was measured in each quadrant (evenly spaced 4 sectors), 12 clock-hour sectors (evenly spaced 12 sectors), and as an average. The quadrant PP-RNFL sector names started with the number 4, while the clock-hour sector names started with the number 12. The average GC-IPL thickness and measurement in each of six sectors (evenly configured sectors centred on the fovea) were collected. Built-in automated segmentation algorithms were used to distinguish each retinal layer. Two investigators (S.Y.L. and Y.H.) independently reconfirmed the image quality, segmentation, and alignment of the measurement window. SS-OCT images with image quality scores > 60 were selected for analysis according to the manufacturer’s recommendation.

The mean absolute difference among three consecutive OCT measurements were calculated as follows:

$$\begin{aligned} & Mean\,absolute\,difference\,of\,image\,quality\,score{\text{:}} \\ & \quad \quad \left( {\left| {IQ1 - IQ2\left| + \right|IQ2 - IQ3\left| + \right|IQ1 - IQ3} \right|} \right)/3 \\ \end{aligned}$$

where IQ_n_—image quality score at the nth measurement.

The subjects were stratified into three groups based on the tertile values of the mean absolute difference of image quality score—LIQD (n = 18), MIQD (n = 19), and HIQD (n = 19). Because subjects in the LIQD group were included in the first third when the mean absolute difference of image quality score was listed in ascending order, they had similar image quality scores among the three consecutive OCT results. In contrast, subjects in the HIQD group showed substantial variation among the three image quality scores because these subjects were the last third subjects.

### Statistical analyses

Analyses of variance and chi-square tests were performed for the comparison of continuous and categorical variables between the groups. A linear mixed model compared the thickness values among the three groups. To determine the repeatability of three consecutive measurements, intraclass correlation coefficients (ICCs) were used. The degree of repeatability was decided according to the ICC value—almost perfect (0.81–1), substantial (0.61–0.8), moderate (0.41–0.6), fair (0.21–0.4), and slight (0–0.2)^22^. To compare the between-group ICC values, the z-score test was used^22–24^. Pearson’s correlation coefficients with and without adjustment of age and sex were used to investigate correlation between the image quality and thickness value. Correlation coefficients were estimated using a linear mixed-effects model to consider three datasets in one individual. All statistical analyses were performed using SAS version 9.4 software (SAS Institute Inc., Cary, NC, USA) by a statistician (H.S.L). Statistical significance was defined as *p* value < 0.05.
